# Design Strategy of Corrosion-Resistant Electrodes for Seawater Electrolysis

**DOI:** 10.3390/ma16072709

**Published:** 2023-03-28

**Authors:** Li Zhao, Xiao Li, Jiayuan Yu, Weijia Zhou

**Affiliations:** Institute for Advanced Interdisciplinary Research (iAIR), School of Chemistry and Chemical Engineering, University of Jinan, Jinan 250022, China

**Keywords:** seawater electrolysis, corrosion-resistant electrode, hydrogen production, protective layer, polyanion layer

## Abstract

Electrocatalytic water splitting for hydrogen (H_2_) production has attracted more and more attention in the context of energy shortages. The use of scarce pure water resources, such as electrolyte, not only increases the cost but also makes application difficult on a large scale. Compared to pure water electrolysis, seawater electrolysis is more competitive in terms of both resource acquisition and economic benefits; however, the complex ionic environment in seawater also brings great challenges to seawater electrolysis technology. Specifically, chloride oxidation-related corrosion and the deposition of insoluble solids on the surface of electrodes during seawater electrolysis make a significant difference to electrocatalytic performance. In response to this issue, design strategies have been proposed to improve the stability of electrodes. Herein, basic principles of seawater electrolysis are first discussed. Then, the design strategy for corrosion-resistant electrodes for seawater electrolysis is recommended. Finally, a development direction for seawater electrolysis in the industrialization process is proposed.

## 1. Introduction

Hydrogen (H_2_), a new kind of high energy density, renewable, and environmentally friendly energy, is an ideal alternative to fossil fuels for energy supply in a zero-carbon emission society [1,2,3,4]. Proton exchange membrane and alkaline water splitting powered by renewable energy are attractive methods for H_2_ production [5]. Economically, the cost of proton exchange membrane and alkaline water electrolysis is less than the cost of direct seawater electrolysis per cubic meter [6]. However, pure water shortage has become a serious issue hindering the large-scale application of the above technology [7,8,9]. Seawater electrolysis is considered a prospective method, as there are abundant seawater resources to provide a large stock for electrolytic water production of H_2_ [8,10,11,12]. Moreover, seawater is an excellent highly conductive electrolyte because of the presence of abundant ions, which also helps to reduce electrolysis cost [13,14,15].

Unfortunately, numerous impurities and corrosive ions in the seawater [16,17,18,19,20] will corrode electrodes during long-term use, resulting in slow cathodic and anodic reactions [21,22]. Therefore, electrodes with high activity and corrosion resistance are essential for seawater electrolysis. Recently, researchers have been committed to designing efficient and stable electrodes for seawater electrolysis [23,24,25]. For example, a unique physical protective layer, or an electrostatic repulsion layer, is constructed on the surface of an electrode to improve stability. Considering the rapid development of seawater electrolysis in recent years, it is urgent to summarize recent progress in the design strategy of corrosion-resistant electrodes for seawater electrolysis.

**Figure 1:**
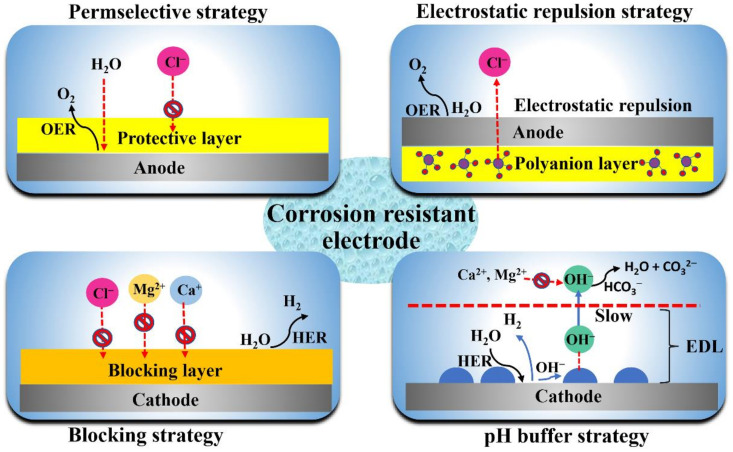
The design strategy of corrosion-resistant electrodes for seawater electrolysis.

Herein, basic principles and existing problems of seawater electrolysis are first reviewed. Then, the design strategy of a corrosion-resistant electrode for seawater electrolysis is recommended (Figure 1). Finally, the development direction of seawater electrolysis in the future is proposed.

## 2. Fundamentals of Seawater Electrolysis

### 2.1. Reaction Mechanism

A traditional water electrolyzer is usually composed of an electrode, electrolyte and a membrane. Water electrolysis mainly involves a hydrogen evolution reaction (HER) and a oxygen evolution reaction (OER), generating gaseous H_2_ and O_2_ on the cathode and anode side, respectively [26,27,28]. Proper electrode application on the cathode and anode can accelerate water splitting. During water electrolysis, the dynamic reaction of an OER (four-electron transfer process) is relatively slow compared to that of a HER (dual-electron transfer process) [29,30,31,32,33,34]. The theoretical potential of overall water electrolysis is 1.23 V [35]. The reaction equations (Equations (1)–(4)) vary among different electrolytes [36].

Acid electrolyte:

Cathode: 2H^+^ + 2e^−^ → H_2_ E^0^ = 0 V (1)

Anode: H_2_O − 2e^−^ → 2H^+^ + 1/2O_2_ E^0^ = +1.23 V (2)

Alkaline electrolyte:

Cathode: 2H_2_O + 2e^−^ → 2OH^−^ + H_2_ E^0^ = −0.83 V (3)

Anode: 2OH^−^ − 2e^−^ → 1/2O_2_ + H_2_O E^0^ = +0.4 V (4)

**Figure 2:**
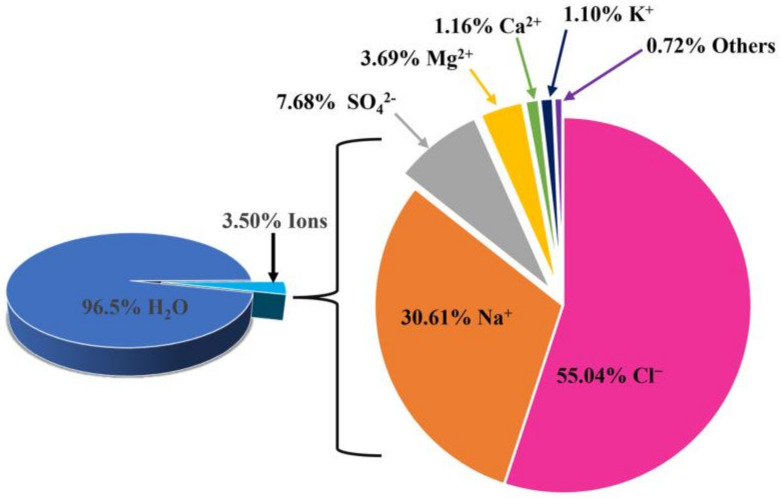
Typical ion composition of seawater. Ref. [38] Copyright 2022, Springer.

The electrolysis of natural seawater is generally analogous to that of conventional water electrolysis, except the two methods use different electrolytes. When the electrolyzer operates in seawater, a large number of active ions, such as Na^+^, Mg^2+^, Ca^2+^ and Cl^−^, in seawater (Figure 2) will seriously hinder water splitting [19,37,38], especially for the OER process. For example, Cl^−^ is considered the main competitor in the oxidation process, owing to its high concentration and standard redox potential, which makes it prone to accrue a chlorine evolution reaction (CER) in the electrochemical process [39,40,41].

### 2.2. Chloride Oxidation

**Figure 3:**
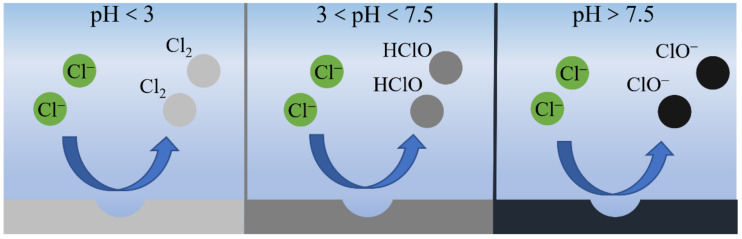
Effect of pH value on chloride ion oxidation process. Ref. [38] Copyright 2022, Springer.

There are various kinds of ions in natural seawater, and Cl^−^ is the main impurity ion. In the process of seawater electrolysis, a high concentration of Cl^−^ will lead to the generation of impurities, such as chlorine or hypochlorite on the anode, which will seriously affect electrolytic activity [42,43,44]. Bennett et al. [45] discovered as early as 1980 that in the process of a cathodic HER with high current density, the anode will produce a large amount of chlorine to form a hypochlorite solution, which seriously affects the production efficiency of oxygen. In addition, Cl^−^ has a very complex reaction process in the electrochemical process, which is influenced by pH value, potential and Cl^−^ concentration. As shown in Figure 3, Strasser et al. [38] found that a CER tended to produce chlorine gas when the pH value of the anode was lower than three. When the pH is greater than 7.5, hypochlorite is more easily generated. As for the pH range of 3~7.5, hypochloric acid is the main product. The reactions in different electrolytes are shown in Equations (5)–(7) [39,46,47,48].

Acid electrolyte:

Anode: 2Cl^−^ − 2e^−^ → Cl_2_ (5)

Anode: 2Cl^−^ + 2H_2_O − 2e^−^ → 2H^+^ + 2HClO (6)

Alkaline electrolyte:

Anode: 2Cl^−^ − 2e^−^ + 2OH^−^ → ClO^−^ + H_2_O (7)

It was also noted that when pH > 7.5, the difference in the potential between hypochlorite formation and the OER will reach its maximum value (480 mV), which indicates that alkaline conditions are beneficial to OER [20,49]. In comparison to an OER, the CER has faster reaction kinetics because it only requires a two-electron transfer process [11,17]. Therefore, designing highly selective electrocatalysts for excellent electrocatalytic activity in alkaline seawater electrolysis is important.

### 2.3. Corrosion and Poisoning of the Electrode

The high concentration of Cl^−^ in seawater will cause a CER, instead of an OER, on the anode to produce chlorine or hypochlorite, which results in the corrosion of the electrode. Cl^−^ can gradually dissolve or directly react with metal, resulting in electrode poisoning and reducing the durability of the electrode [50,51,52]. Furthermore, Mg^2+^, Ca^2+^ and Na^+^ can also cause strong corrosion of the electrode. During the reaction, Mg^2+^, Ca^2+^ and Na^+^ interact with, and replace some cations on, the electrode, thus affecting the activity and stability of the electrode [39,53,54,55]. What is more serious is that, once inactive and insoluble precipitates are deposited on the electrode surface, the catalytic site of the electrode will be blocked, leading to catalyst poisoning or accelerated aging [56,57,58,59]. Furthermore, the local pH value of the electrode surface will vary significantly at high current densities [60,61]. In the process of electrocatalysis, the surface is acidic at the anode side and alkaline at the cathode. When pH > 9, precipitates will be formed on the cathode side to block the active site of the cathode and reduce catalytic activity [58,62,63,64].

### 2.4. Evaluation Metrics for Electrocatalytic Performance

To assess the electrocatalytic performances of different catalysts, several significant parameters, including overpotential, Tafel slope, electrochemical active area and stability, etc., need to have certain values [29]. These parameters can also help us understand the thermodynamics and kinetics of electrocatalytic reactions.

#### 2.4.1. Overpotential

Overpotential is an important parameter for detecting the electrocatalytic activity of electrodes. It is originated from the intrinsic kinetic barriers or solution/contact resistances of electrodes. Overpotential is the difference between the potential that achieves a certain current density and the potential determined by the thermodynamics of an electrochemical reaction. At present, the overpotential for achieving 10 mA cm^−2^ is the most important value for evaluating the activity of an electrode [65,66,67,68].

#### 2.4.2. Tafel Slope

The reaction kinetics for an HER/OER can be revealed by a Tafel slope. The Tafel slope is the polarization of the electrode that reflects the blocked electrode process. The smaller the Tafel slope, the faster the reaction kinetics. A Tafel plot can be obtained from the corresponding linear sweep voltammetry (LSV) curve, which is expressed as the equation η = a + b log (j) (η is overpotential, j is the current density, and b is the Tafel slope) at the linear region. The value of the Tafel slope is obtained by fitting the linear regions [69,70].

The Tafel slope can give insight into the reaction mechanism of an HER/OER. Taking an HER as an example, if the value of the Tafel slope is between 30 and 40 mV dec^−1^, the reaction mechanism is determined using the Volmer–Tafel mechanism, and the rate-determining step (RDS) is the Tafel step. If the value is >40 mV dec^−1^, the reaction mechanism is determined using the Volmer–Heyrovsky mechanism. It is worth noting that if the value is between 40 and 120 mV dec^−1^, the RDS is the Heyrovsky step, and if the value is >120 mV dec^−1^, the RDS is the Volmer step.

#### 2.4.3. Electrochemical Surface Area

For comparing the performance of different electrodes conveniently in the same electrocatalytic reaction, the electrochemical surface area (ECSA) is proposed. The active site of a traditional electrode is considered to be the external surface in contact with the electrolyte, and geometric area can, thus, be considered to be approximately equal to the ECSA. However, an ideal ECSA is the contact area between the active site and the electrolyte. Because many active sites are not a simple layer, it is difficult to measure them by physical means. Therefore, there are many electrochemical approaches to simulate and calculate the ECSA. At present, there are many ways to characterize the number of active sites, such as atomic calculation or quantitative ECSA using a cyclic voltammetry (CV) test [71,72].

#### 2.4.4. Long-Term Stability

Under the determined redox reaction environment, the structure and organization of the electrode will inevitably change significantly after long-term operation. For example, if the electrode is attacked by bubbles and falls off during operation, or is damaged by some corrosive ions, it may lead to serious deterioration of catalytic performance. Therefore, it is very important to ensure that the electrode is not disturbed by external factors. The long-term stability is a very significant parameter for estimating the ability of the catalyst to retain its original performance after a period of time. Stability can be studied by continuous CV or chronopotentiometric/hourly amperometry [73].

The performance evaluation of recently reported advanced catalysts for seawater electrolysis is summarized in Table 1.

### 2.5. Key Aspects for Designing a Corrosion-Resistant Electrode

Conventional electrocatalysts (noble and non-noble metals, transition metal sulfides, etc.) possess outstanding activity in seawater electrolysis [57,87]. Exposing more active sites [74,84,85,88,89] and adjusting the adsorption energy of reaction intermediates on the catalyst surface can reduce the reaction potential energy [75,90,91,92]. These methods optimize the electrode structure to reduce the reaction obstacles for improving the reaction activity [93,94], rather than directly isolating corrosive ions and impurities in seawater from the electrode. However, active sites may be corroded or blocked at the electrochemical reaction due to complex ions in seawater. Therefore, on the basis of a traditional electrode, a seawater-based electrode needs to be highly stable and corrosion resistant. Several effective strategies have been adopted for promoting the corrosion resistance of electrodes for seawater electrolysis.

## 3. Design Strategies for a Corrosion-Resistant Electrode

As mentioned above, the electrochemical reaction process of seawater electrolysis will cause side reactions due to various impurities in seawater, and, thus, disturb the seawater electrolysis process. One of the main challenges in seawater electrolysis is to avoid a CER, because a CER not only produces chlorine gas or hypochlorous acid that competes with the OER of the anode but also corrodes the electrode to reduce its stability and activity [80,81]. Therefore, corresponding design strategies, including coating a physical protection layer or using electrostatic repulsion to block or repel Cl^−^, are effective means.

### 3.1. Design Strategies for a Corrosion-Resistant Anode

#### 3.1.1. Permselective Strategy

Seawater contains a large amount of Cl^−^, which will cause a CER on the anode, causing the anode to corrode, and seriously affecting the service life and electrolytic activity of the electrode. So far, many investigations of anticorrosion design have focused on the preparation of a protective layer [95,96].

**Figure 4:**
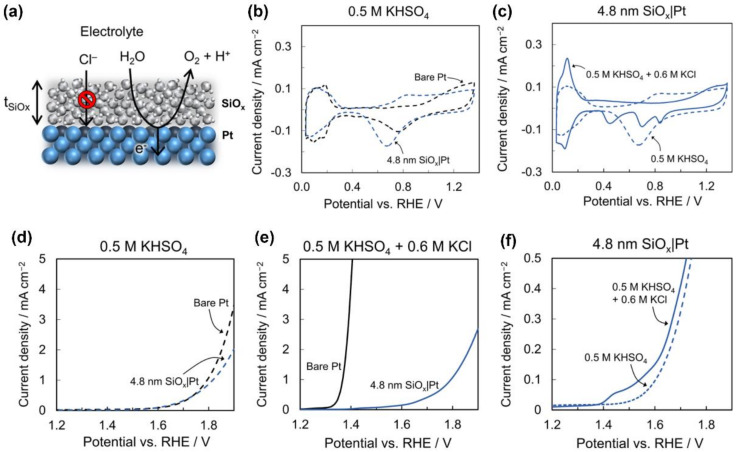
(**a**) Schematic diagram of the SiO_x_|Pt electrode. (**b**) CV for 2 electrodes in 0.5 M KHSO_4_. (**c**) CV and (**f**) for 4.8 nm SiO_x_|Pt in different electrolytes. LSV for 2 electrodes in (**d**) 0.5 M KHSO_4_ and (**e**) 0.5 M KHSO_4_ + 0.6 M KCl. Reproduced with permission. Ref. [97] Copyright 2021, American Chemical Society.

Amar et al. [97] prepared a nanoscale ultra-thin silicon oxide (SiO_x_) inert layer deposited on a planar Pt thin film electrode using photochemical technology to reject the transport of Cl^−^ and prevent the occurrence of a CER (Figure 4a). To understand the ability of a covering layer for inhibiting a CER, a series of electrochemical tests were conducted. As shown in Figure 4b, the H_upd_ signal for a 4.8 nm SiO_x_|Pt electrode is only 4% lower than the signal for an electrode made of bare Pt, indicating that the Pt surface at the buried interface between Pt and SiO_x_ has the catalytic activity. Compared with bare Pt, the response of the presence of Cl^−^ on a 4.8 nm SiO_x_|Pt electrode is quite different (Figure 4c). As shown in Figure 4d, the starting potential of an OER on a 4.8 nm SiO_x_|Pt and a bare Pt electrode is almost the same in chlorine-free supported electrolyte at an acidic pH. In the presence of 0.6 M Cl^−^, the initial potential of a CER on bare Pt was 270 mV lower than that of an OER in the supported electrolyte (Figure 4e). In the 0.6 M KCl + 0.5 M KHSO_4_ supported electrolyte, a 4.8 nm SiO_x_|Pt electrode had almost no CER oxidation peak at 1.35 V, indicating that the SiO_x_ coating hindered the transmission of Cl^−^ (Figure 4f). The above results indicated that the inert layer could effectively inhibit a CER by hindering the transfer of Cl^−^ to the electrode’s buried interface, while permitting the required an OER to occur there. Finally, by optimizing the structure of the SiO_x_ layer, researchers added fixed anionic charges to the electrostatic repulsion of Cl^−^ to further improve the chlorine resistance of the SiO_x_ coating.

**Figure 5:**
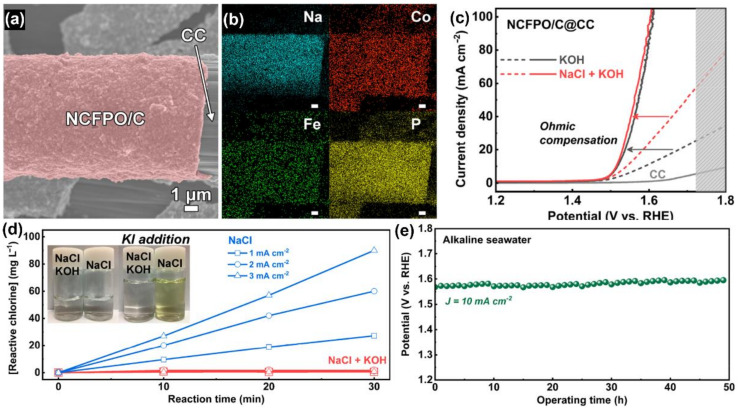
(**a**) FESEM and (**b**) SEM-EDS elemental mapping of NCFPO/C@CC. (**c**) LSV of NCFPO/C@CC in two different electrolytes. (**d**) Time-dependent reactive chlorine concentration profiles. (**e**) Chronopotentiometric test in alkaline seawater. Reproduced with permission. Ref. [82] Copyright 2020, American Chemical Society.

In addition, a carbon layer can also be used as the protective layer to promote the stability and corrosion resistance of the electrode. Hee et al. [82] developed carbon coated cobalt sodium pyrophosphate nanoparticles (NCFPO/CNPs) via a series of heat treatment methods. It was shown that NCFPO/CNPs were evenly coated on the carbon cloth surface (Figure 5a,b). As shown in Figure 5c, OER activities of NCFPO/C@CC in 0.1 M KOH and 0.5 M NaCl + 0.1 M KOH electrolytes were similar, demonstrating that Cl^−^ had little influence on the electrocatalytic performance. To further prove the ability of an electrode to inhibit a CER in an alkaline saline solution, iodide titration was used to determine whether there was active chlorine. In fact, the color changed in an NaCl solution, but not in an NaCl+KOH solution (Figure 5d). This showed that the electrode did not produce active chlorine in the alkaline saline solution, which effectively inhibited a CER. In the process of an OER, the hydroxide evolved on the surface of the NCFPO/C NPs acted as the active site of oxygen generation, which improved the catalytic activity of an OER. Moreover, the carbon layer coated on the electrode surface could effectively isolate the corrosive Cl^−^ in the seawater, so that active chlorine was not produced, which led to electrode corrosion. In the constant current stability test, the required voltage did not change basically, demonstrating the electrode’s stability (Figure 5e).

#### 3.1.2. Electrostatic Repulsion Strategy

Although the physical protective layer can prevent the corrosion and improve the stability of the electrode, it may overlay active sites and affect the catalytic performance. To avoid this problem, the insertion of a polyanion layer as an electrostatic repulsion layer into the interior of the active material can not only effectively prevent the adsorption of Cl^−^ but also not affect the exposure of active sites.

**Figure 6:**
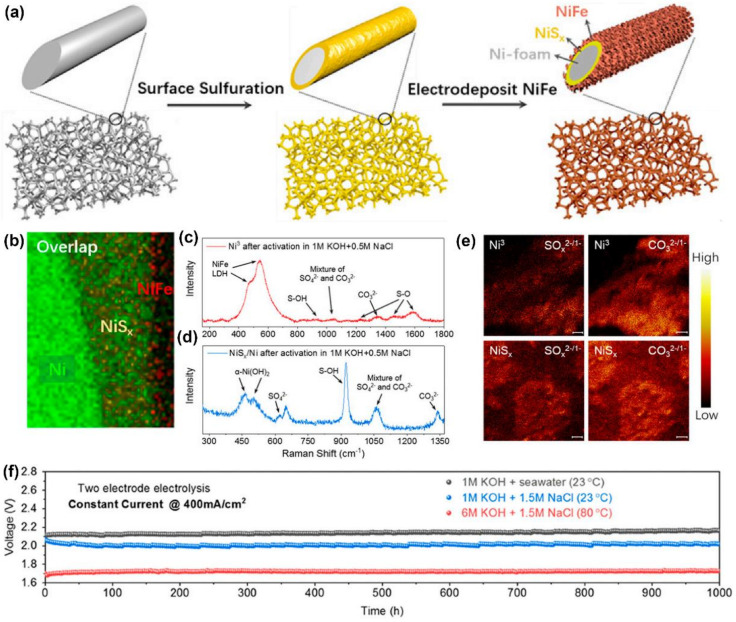
(**a**) Schematic diagram of the synthetic process of NiFe/NiS_x_-Ni foam. (**b**) Elemental mapping of NiFe/NiS_x_-Ni foam (cross-section). Raman spectra of (**c**) NiFe/NiS_x_-Ni foam and (**d**) NiS_x_/Ni after 12 h activation. (**e**) TOF-SIMS mapping of NiS_x_/Ni after activation. (**f**) Results from 1000 h tests of the seawater-splitting electrolyzer at 400 mA cm^−2^. Reproduced with permission. Ref. [83] Copyright 2019, National Academy of Sciences.

Kuang et al. [83] developed a low-cost electrode (NiFe/NiS_x_-Ni foam) for seawater electrolysis without chloride corrosion. The NiS_x_ covering layer on Ni foam was synthesized by a solvothermal process. Then, NiFe hydroxide was electrodeposited by reducing the nitrate from a mixed solution of nickel and iron nitrate (Figure 6a). The image of cross-section element mapping showed that a NiS_x_ layer (about 1 to 2 μm) was coated on the Ni foam, and a NiFe layer (about 200 nm thick) was formed on the NiS_x_ layer (Figure 6b). After anodic activation, about 0.3 V overpotential was added to the NiFe/NiS_x_-Ni foam anode to reach 400 mA cm^−2^ in an alkaline simulated seawater electrolyte, which was much lower than that used for the oxidation of chloride to hypochlorite (0.49 V). Stability tests showed that the introduction of a NiS_x_ polyanion layer could greatly improve the ability to resist chlorine corrosion. Raman spectroscopy revealed that the activation/passivation treatment of NiFe/NiS_x_-Ni foam resulted in transient etching; consequently, the polyanions were passivated through the layer of the anode (Figure 6c,d). The excellent OER performance and corrosion resistance of NiFe/NiS_x_-Ni foam to Cl^−^ was derived from the sulfate and carbonate intercalated NiFe hydroxide catalyst layer and the underlying sulfate-rich anodized NiS_x_ layer. The activated/passivated NiFe/NiS_x_-Ni foam anode was assembled with an active HER cathode (Ni-NiO-Cr_2_O_3_) to form a two-electrode alkaline seawater electrolyzer. This seawater electrolyzer could operate stably under high temperatures and concentrated base conditions (typically used in industry), only needing 1.72 V to achieve 400 mA cm^−2^ in 6 M KOH + 1.5 M NaCl at 80 °C for over 1000 h (Figure 6e,f).

Similarly, Li et al. [86] synthesized an interlayer of Ni_3_S_2_-MoS_2_-Ni_3_S_2_@Ni (NMN-NF) as an efficient OER electrode for seawater electrolysis. In the process of activation, the NiS_x_ layer formed polyanions dominated by sulfate ions. When the negative polyatomic anion (SO_4_^2−^) was embedded in the anode, it repelled the Cl^−^ in the seawater and did not allow it to reach the surface of the electrode; therefore, a sulfate-rich Ni_3_S_2_ sandwich structure was crucial to inhibiting the corrosion of Cl^−^ in the OER process. Operating in alkaline seawater for more than 100 h, the electrode remained high catalyst active, which shows almost no attenuation during the electrolysis process.

**Figure 7:**
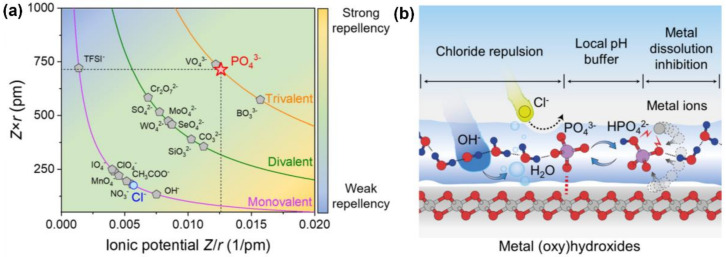
(**a**) Repellency between common anions and Cl^−^. (**b**) Schematic diagram of the anticorrosion mechanism. Reproduced with permission. Ref. [98] Copyright 2022, Elsevier.

To explore the resistance mechanism for Cl^−^ using polyanions and the rejection effect of different polyanions, Yu et al. [98] inserted corresponding anionic additives in the preparation process of NiFe-LDH and obtained a series of comparable samples. The electrochemical test showed that PO_4_^3−^ had ideal ionic potential. The ratio of Z/r endowed PO_4_^3−^ with a superior ability to repel Cl^−^ (Figure 7a). Moreover, the PO_4_^3−^ anion had high electrochemical stability and large electrostatic potential (Figure 7b), which made the adsorbed PO_4_^3−^ form a soft “semi-permeable layer”. The water on the surface repelled chlorine instead of hindering the diffusion of OH^−^, greatly improving the performance of the electrode.

### 3.2. Design Strategies for a Corrosion-Resistant Cathode

Compared to the anode, the cathode will not only be corroded by chlorine or hypochlorite but will also encounter passivation and deactivation caused by the deposition of insoluble precipitation on the electrode surface. Extensive cations, such as Ca^2+^ and Mg^2+^, will form hydroxide and deposit on the cathode, blocking and corroding the catalytic active site, and seriously affecting the activity and stability of the electrode [45,76,99,100,101]. There are several effective strategies to solve these problems that have been reported in recent works.

#### Blocking Strategy

Covering the cathode in an anticorrosion protective barrier layer outside of the active material has been demonstrated to limit the contact between the active material and harmful ions, thus enhancing its long-term stability.

**Figure 8:**
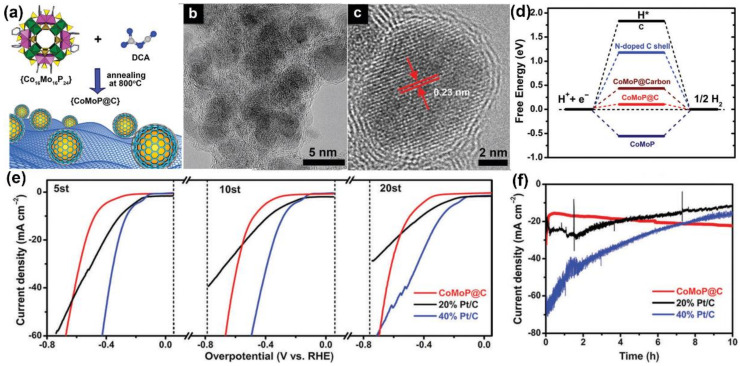
(**a**) Preparation process diagram of the CoMoP@C. (**b**,**c**) HRTEM images of CoMoP@C. (**d**) The free-energy diagram of the HER. (**e**) LSV plots of three catalysts in natural seawater. (**f**) I-t curves of three catalysts in seawater at overpotential (500 mV) for 10 h. Reproduced with permission. Ref. [77] Copyright 2017, Royal Society of Chemistry.

Utilizing polyoxometalate (Co_16_Mo_16_P_24_) and dicyandiamide (DCA) as precursor, Ma et al. [77] fabricated CoMoP nanocrystals covered by a small amount of N-doped carbon shells (CoMoP@C) by pyrolysis under a nitrogen atmosphere at 800 °C (Figure 8a). Using a transmission electron microscope, it was observed that the morphology structure of CoMoP@C exhibited a typical core-shell structure (Figure 8b,c). The CoMoP was wrapped with several layers of graphite carbon, which could effectively prevent the corrosion of harmful ions. The doping of N increased the electron density in the graphite carbon shell and enhanced the HER activity, as confirmed by a density functional theory calculation (Figure 8d). In the actual seawater test, researchers found that the performance of CoMoP@C decreased by less than 5% after 20 cycles, while that of Pt/C rapidly decreased by 40% (Figure 8e). To further study the impact of N-doped carbon shells on stability, the i-t testing of CoMoP NPs and CoMoP@C in seawater electrolysis was tested (Figure 8f). The results indicated that the CoMoP@C remained stable for 10 h without any change in current density. In the absence of N-doped carbon shell protection, the current density of CoMoP NPs decreased by more than 75% within 3 h. The above series of experiments showed that the remarkable stability of the catalyst was attributed to the carbon layer, protecting the catalyst from toxicity and corrosion during seawater electrolysis.

Similarly, Bu et al. [78] prepared a unique heterostructure (NiMo@C_3_N_5_) consisting of 1D NiMo as a core and 2D C_3_N_5_ as a shell by a hydrothermal reaction method. The heterojunction, as an electrocatalyst for an HER, showed excellent performance in natural seawater. The excellent core-shell structure made the C_3_N_5_ nano-layer tightly wrap the NiMo nanorods. The C_3_N_5_ shell acted as a protect layer to prevent the NiMo from corrosion and toxicity caused by chemical impurities in seawater. Meanwhile, the C_3_N_5_ shell had no effect on the electrochemical reaction on the NiMo core. In addition, NiMo@C_3_N_5_ showed stable HER performance, with a faraday efficiency in seawater electrolysis of up to 94.8%.

**Figure 9:**
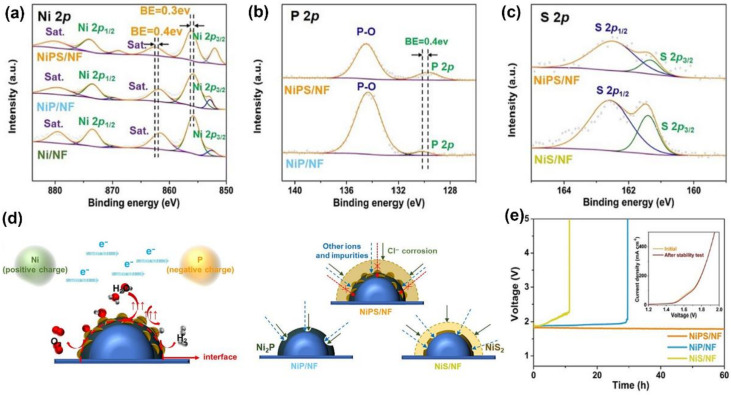
XPS spectra of samples for (**a**) Ni 2p, (**b**) P 2p and (**c**) S 2p. (**d**) Illustration of intensive performance of NiPS/NF for water electrolysis. (**e**) Long-term stability of samples at 200 mA cm^−2^. Reproduced with permission. Ref. [87] Copyright 2022, Elsevier.

In addition to coating a carbon layer near the catalyst as a protective layer, the application of a polyanion layer to repel Cl^−^ in contact with the electrode surface is also an effective strategy. For instance, Wang et al. [87] used an electrodeposition process to grow a Ni_2_P/NiS_2_ microsphere on Ni foam as the electrode for an HER. XPS analysis (Figure 9a–c) showed that the electronic interaction of nickel phosphide and sulfide caused the charge to be redistributed on the coupling interface, providing the Ni–P bond with more favorable covalent bond properties and making it easier for it to adsorb H^*^ and H_2_O, thus promoting the HER activity. More importantly, the sulfide could be activated to form multivalent anions, which could block and repel Cl^−^ on the electrode surface, in order to improve the stability of Ni_2_P/NiS_2_ (Figure 9d). In the natural alkaline seawater electrolyte, Ni_2_P/NiS_2_ could operate for more than 60 h without the obvious attenuation of current density (Figure 9e), which demonstrated excellent seawater electrolysis stability.

### 3.3. pH Buffer Strategy

Inhibiting the formation of insoluble hydroxide precipitates on the cathode surface is essential to maintain HER activity and stability. Based on classic acid base theory, hard acid can be preferentially bound to a hard base. Recently, Guo et al. [102] introduced a Lewis acid layer (e.g., Cr_2_O_3_) on CoO_x_ to dynamically enhance the activity and stability of the cathode for an HER. Using this layer to split H_2_O molecules and capture the generated hydroxyl anion, they could artificially create an alkaline microenvironment. Such locally generated alkalinity was conducive to the kinetics of the reaction between the two electrodes, and created an abundance of OH^−^ to help resist the arrival of Cl^−^ an effectively inhibited a CER. In addition, the strong combination of OH^−^ and the Lewis acid layer significantly reduced the OH^−^ captured by Mg^2+^ and Ca^2+^ to alleviate the formation of precipitation. The stability was maintained for over 100 h at 500 mA cm^−2^, which was similar to that of a PEM electrolytic cell circulating in high-purity water.

## 4. Conclusions and Outlook

Due to abundant seawater reserves and reasonable economic feasibility, the above strategies are promising methods to achieve large-scale electrolytic hydrogen production from seawater. However, because seawater contains a complex ionic composition, seawater electrolysis technology faces some obstacles, such as low efficiency, lack of long-term stability, etc. In this review, the basic principles and challenges of seawater electrolysis are discussed. Then, the design strategies for a corrosion-resistant electrode for seawater electrolysis have been summarized, including a permselective strategy, electrostatic repulsion strategy, blocking strategy and pH buffer strategy. Although great progress has been made in seawater electrolysis hydrogen production technology, there is still a long way to go before large-scale industrialization. Therefore, we propose some suggestions to promote the future development in this field.

### 4.1. Efficient Screening Electrocatalysts

At present, theoretical calculation is widely applied in the study of electrocatalysts. Important progress has been made in predicting the activity of electrocatalysts and assisting in the design of high-performance electrocatalysts. Future works may look at the combination of experimental study and theoretical calculation. Furthermore, theoretical calculation will be used to simulate a more actual reaction mechanism, thus narrowing or eliminating the difference between existent experimental study and theoretical calculation.

### 4.2. Understand the Reaction Mechanism In-Depth

At present, the mechanisms of a series of redox reactions in the seawater electrolysis process are still controversial. An understanding of the major electrocatalytic and interfacial reactions of various ions facilitates the control of reaction processes and the design of catalytic performance. Herein, we recommend constructing advanced in situ measurement techniques for the instantaneous analysis of important intermediates and real active components. Combining the existing operational techniques with sophisticated simulations will provide a solid method to decipher structural evolution and actual reaction mechanisms during the reaction.

### 4.3. Establish a Standard Criteria System

The application of standardized systems to evaluate the performance of new electrocatalysts used in seawater electrolysis will benefit the community. The components of natural seawater are complex, and it is important to use standardized electrolyte composition as a benchmark for new electrocatalysts. For buffered seawater, similar standards should be applied for the clear definition of the nature and concentration of buffered species.

## Figures and Tables

**Table 1:** Recently reported advanced catalysts for seawater electrolysis.

Catalysts	Electrolyte	η@j (mV@mA cm^2^)	Tafel Slope (mV dec^−1^)	ECSA (mF cm^−2^)	Stability (h)	Refs.
HER						
Co_80_B_5_P_15_	1 M KOH	42@10	39.8	8.26	20	[26]
Mn–NiO–Ni/Ni-F	Seawater	200@35	121.0	/	50	[46]
Pt-Te-MoS_2_	1 M KOH	52@10	62.3	/	8	[74]
Cu_2_S@Ni	1 M NaOH + 0.5 M NaCl	200@530	95.1	/	150	[75]
Ni-MoO_3_	Seawater	412@10	133.0	13.24	24	[76]
CoMoP@C	Seawater	450@10	49.7	37.60	10	[77]
NiMo@C_3_N_5_	Seawater	486@10	68.3	14.60	10	[78]
OER						
SSFF@NiFe LDH	1 M KOH	198@10	31.6	3.63	10	[34]
B-Co_2_Fe LDH	1 M KOH + Seawater	205@10	39.2	/	100	[79]
S-Cu_2_O-CuO NDLs	1 M KOH + Seawater	450@1000	45.0	/	100	[80]
CoP_x_@FeOOH	1 M KOH + Seawater	337@500	37.6	/	80	[81]
Na_2_CoP_2_O_7_/C	1 M KOH + 0.5 M NaCl	480@100	47.0	53	100	[82]
NiFe/NiS_x_/Ni	1 M KOH + 0.5 M NaCl	510@400	/	/	1000	[83]
Bifunction						
Ni_3_S_2_@Ni_2_P/MoS_2_	1 M KOH	175@10	46.0	4.50	40	[44]
FeNi OH-100	1 M KOH	236@50	48.9	66.86	25	[62]
NiSe@Co_0.85_Se/NF	1 M KOH	258@10	50.0	5.61	20	[84]
NiFe-LDH/FeOOH	1 M NaOH + 0.5 M NaCl	274@100	69.8	/	105	[85]
Ni_3_S_2_-MoS_2_-Ni_3_S_2_@Ni	1 M KOH + 0.5 M NaCl	188@100	48.0	15.20	100	[86]
Ni_2_P/NiS_2_	1 M KOH + Seawater	391@500	23.0	75.10	48	[87]

## Abbreviations

PEM	Proton exchange membrane
HER	hydrogen evolution reaction
OER	oxygen evolution reaction
CER	chlorine evolution reaction
LSV	linear sweep voltammetry
CV	cyclic voltammetry
ECSA	electrochemical surface area
FESEM	field emission scanning electron microscopy
HRTEM	high resolution transmission electron microscope
SEM	scanning electron microscope
EDS	energy dispersive spectroscopy
XPS	X-ray photoelectron spectroscopy
DCA	dicyandiamide
TEM	transmission electron microscope
